# Tendon Repair Is Compromised in a High Fat Diet-Induced Mouse Model of Obesity and Type 2 Diabetes

**DOI:** 10.1371/journal.pone.0091234

**Published:** 2014-03-21

**Authors:** Michael A. David, Khyrie H. Jones, Jason A. Inzana, Michael J. Zuscik, Hani A. Awad, Robert A. Mooney

**Affiliations:** 1 Department of Pathology and Laboratory Medicine, University of Rochester Medical Center, Rochester, New York, United States of America; 2 Department of Biomedical Engineering, University of Rochester, Rochester, New York, United States of America; 3 Center for Musculoskeletal Research, University of Rochester Medical Center, Rochester, New York, United States of America; University of Sheffield, United Kingdom

## Abstract

**Introduction:**

The obesity epidemic has resulted in a large increase in type 2 diabetes (T2D). While some secondary complications of T2D are well recognized and their cellular and molecular mechanisms are defined, the impact of T2D on the musculoskeletal system is less understood. Clinical evidence suggests that tendon strength and repair are compromised. Here, a mouse model of obesity and T2D recapitulates the deleterious effects of this condition on tendon repair.

**Methods:**

Male C57BL/6J mice at 5 weeks of age were placed on a high fat (HF)(60% kcal) or low fat (10% kcal) diet for 12 weeks. The flexor digitorum longus (FDL) tendon was then injured by puncturing it with a beveled needle. Progression of FDL tendon healing was assessed through biomechanical and histological analysis at 0, 7, 14 and 28 days post-injury.

**Results:**

HF-fed mice displayed increased body weight and elevated fasting glucose levels, both consistent with T2D. No differences in biomechanical properties of the uninjured FDL tendon were observed after 12 weeks on HF versus lean diets, but decreased maximum force in uninjured tendons from HF-fed mice was observed at 24 weeks. Following puncture injury, tendons from HF-fed mice displayed impaired biomechanical properties at day 28 post injury. In support of defective repair in the HF-fed mice, histological examination of the injury site showed a smaller area of repair and lower cell content in the repair area of HF-fed mice. Insulin receptors were expressed in most cells at the injury site regardless of diet.

**Discussion:**

The HF-diet mouse model of obesity and T2D reproduces the impaired tendon healing that is observed in this patient population. The exact mechanism is unknown, but we hypothesize that a cellular defect, perhaps involving insulin resistance, leads to decreased proliferation or recruitment to the injury site, and ultimately contributes to defective tendon healing.

## Introduction

The epidemic of obesity, in part due to the consumption of a high-fat Western diet, has resulted in a marked increase in type 2 diabetes [1]. Diabetes mellitus is a chronic endocrine disorder characterized by elevated blood glucose levels. In type 2 diabetes, the development of insulin resistance is a critical component of the dysregulation of blood glucose [2]. According to the National Diabetes Fact Sheet 2011, the Centers for Disease Control (CDC) reports that a total of 18.8 million individuals are diagnosed with diabetes in the United States with another 7.0 million undiagnosed [3]. Greater than 90% of these cases are type 2 diabetics with 80–90% of type 2 diabetics being obese [4]. Statistics also indicate that the total estimated medical costs of diabetes in 2012 has reached $245 billion [5]. Though the complications of diabetes include heart attack and stroke, kidney disease, hypertension, amputations and blindness, the effects on the skeletal system, and particularly tendon injury and repair, are poorly understood [6], [7].

Tendons transmit contractile forces from muscle to bone, resulting in joint movement. Following injury, tendon healing occurs in three overlapping phases: inflammatory, proliferative, and remodeling [8]. In the inflammatory phase, vasculature that surrounds the injury site contributes to the formation of a hematoma [9], [10]. Immune cells migrate to the wound site where cellular debris, necrotic materials, and foreign body matter are engulfed by phagocytosis [9], [11]. This is also accompanied by an increase in cell number, fibronectin, glycosaminoglycan, water, and collagen 3, which collectively stabilize the newly formed provisional matrix [12]. Most importantly, tenocytes are recruited to the wounded area and proliferate [8], [13]. The proliferative phase is characterized by profuse synthetic activity directed by macrophages and tenocytes. Macrophages release growth factors, while tenocytes deposit a matrix composed of collagen 3, which peaks during this stage [8], [11], [12]. During the remodeling phase, collagen 3 synthesis decreases while collagen 1 synthesis dominates [8], [14], [15]. This is accompanied by a decrease in cellularity, matrix synthesis is reduced, and the extracellular matrix becomes more aligned [9], [16]. Collagen 1 fibers are organized longitudinally along the tendon axis and are responsible for the mechanical strength of the regenerated tissue [9]. Lastly, the fibrous repair tissue gradually converts to scar tissue [11].

The strength and recovery of tendons from injury in the presence of diabetes have not been extensively assessed. Previous research has suggested, however, that diabetes compromises the biomechanical properties of injured tendons that are healed, such as the Achilles and those in the rotator cuff, by impairing the healing process [17]–[19]. To investigate impaired tendon repair and wound healing as a consequence of type 2 diabetes, an injury and repair model was developed for the flexor digitorum longus (FDL) tendon in the classic high fat diet-induced obese, diabetic mouse. We tested the hypothesis that metabolic dysfunction in this type 2 diabetes model would compromise the biomechanical properties and repair mechanisms of the FDL tendon following injury.

## Materials and Methods

### Ethics Statement

All handling of mice and associated experimental procedures were reviewed and approved by the University Committee on Animal Resources at the University of Rochester Medical Center.

### Animals

Male C57BL/6 mice purchased from Jackson Laboratories were housed 5 per cage in a microisolator room on a 12 hr light/dark cycle at the University of Rochester Medical Center. Mice were placed on a high fat (HF)(fat 60 kcal%, carbohydrate 20 kcal%, protein 20 kcal%; D12492) or low fat (fat 10 kcal%, carbohydrate 70 kcal%, protein 20kcal%; D12450B) diet at 5 weeks of age (Open Source Diets, Research Diets Inc., New Brunswick, NJ). Mice remained on the respective ad libitum diets for 12 weeks. Body weight and fasting blood glucose were measured monthly but reported here at 12 weeks and again at tissue harvest. For tail vein blood collection, mice were placed under isoflurane anesthesia in the morning following an overnight fast. Glucose measurements were made using a One Touch glucose meter (Lifescan, Inc.). Mice were either placed in the tendon injury study or fed a lean diet for an additional 12 weeks in the absence of an injury.

### Surgical Procedure

At the time of surgery, mice were anesthetized via an intraperitoneal injection of ketamine (60 mg/kg body weight) and Xylazine (4 mg/kg body weight). Surgeries were performed aseptically with the aid of a 2× dissecting microscope. Briefly, a longitudinal incision was made from the right hind paw to the talus. Once the flexor digitorum longus (FDL) tendon was exposed with forceps, a spatula was placed under the tendon. Using another set of forceps opened to a fixed spacing of 3 mm, the tendon was lifted vertically, providing a gap between the tendon and spatula. The tendon was punctured using a ¾ inch long, beveled 23, 25, or 27 gauge needle that was passed through the tendon at a 45 degree angle while centered medially and laterally. The contralateral left limb was sham-operated with the uninjured but exposed tendon subsequently serving as an experimental control. Progression of the FDL tendon healing process was assessed through biomechanical and histological analysis at 0, 7, 14 and 28 days post-injury.

### Biomechanical Testing

For biomechanical testing, tissue surrounding the entire length of the tendon was removed, from the tarsal tunnel to the myotendinous junction. The proximal end of the FDL tendon was blunt dissected from the myotendinous junction, retaining a small amount of muscle attached to the end of the tendon. At the distal end, the hind paw was isolated together with the FDL tendon. Tendon and the attached hind paw were placed in 0.9% saline and stored at −20°C until testing.

At the time of testing, each tendon was thawed at room temperature for 30 min. The proximal end of the tendon was affixed to tape using cyanoacrylate glue and allowed to dry for 28 min while saturating the tendon with 1× phosphate buffered saline (PBS) to prevent dehydration. The initial length of the tendon for post analysis was measured from the footpad to the tendon/glue interface in all cases except the 12 week time point which was measured from the toes to the interface. The tendon was then loaded onto a uniaxial tensile testing machine, Instron DynaMite 8841 (Instron, Norwood, MA) by clamping the proximal end at the glue interface and the distal end at the paw [20]. Tensile force was applied to create a distraction rate of 30 mm/min until failure. Force-displacement graphs were plotted and the maximum force, work to maximum force and stiffness were calculated.

### Histology

Tendons to be used for histologic analysis were isolated via a procedure that was similar to that utilized for biomechanical testing. In this case, the proximal end was freed of muscle and the distal end was separated from the paw at the tarsal tunnel. Tendons were affixed to cardboard coated with biopsy-grade bio-wrap (Leica Microsystems Inc., Buffalo Grove, IL) and suspended in 10% neutral buffered formalin overnight. Tissues were then dehydrated and embedded in paraffin. Five micron sagittal sections were cut beginning on the lateral side and progressing toward the medial side of the tendon. Sections through the injury site were stained with hematoxylin-eosin and picrosirius red.

### Immunohistochemistry

Immunohistochemistry was performed as described previously [21]. Briefly, an avidin-biotin peroxidase system (Vector Lab, Burlingame, CA, USA) was used to detect anti-insulin receptor rabbit polyclonal antibody (#SC-711: Santa Cruz Biotechnology, Inc., Santa Cruz, CA). Reactions were then visualized with diaminobenzidine (DAB) as substrate (Vector Lab, Burlingame, CA, USA). The sections were counterstained with hematoxylin. All staining procedures were performed as described by the manufacturer.

### Quantification of Repair Tissue

Area of tendon that was actively repairing and remodeling was quantitated on hematoxylin-eosin-stained sections as a fraction of total tendon area using OsteoMeasure software (OsteoMetrics, Decatur, GA) at 5× magnification. Similarly, cell densities in regions of repair were quantified by averaging cell counts in five representative sites within each repair region.

### Statistical Analysis

For data analysis of two groups, student's t-tests were performed (α = 0.05), while Analysis of Variance (ANOVA) with Bonferroni's post-hoc multiple comparisons (α = 0.05) were used when comparing three or more groups. Statistical analysis was done using Prism GraphPad 4.0 statistical software.

## Results

### Developing a mouse tendon injury model

**Figure 1:**
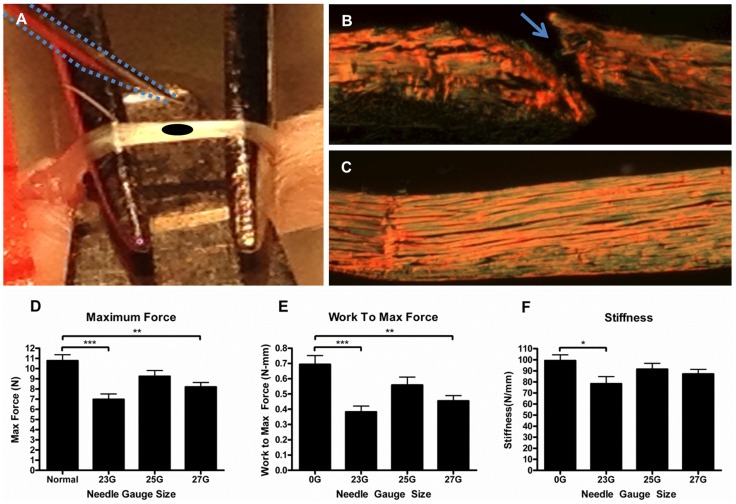
Murine “Stab Injury” model. A 23-gauge needle (outlined by blue dotted line) approaches the surface of an exposed and elevated FDL tendon (A) prior to creating the stab injury defect (depicted by black oval). Picrosirius red stained longitudinal sections of (B) injured (blue arrow) and (C) uninjured, sham operated FDL tendons are visualized under polarized light (5× magnification). Note that the tissue section in (B) is oriented through the center of the stab injury. Maximum force (D), work to maximum force (E) and stiffness (F) were measured in isolated FDL tendons immediately following a puncture injury produced by various needle gauge sizes. Results are represented as mean ± SEM (n = 7–9). *** p<0.001, ** p<0.01 and * p<0.05.

While clinical evidence has indicated that the tendons of diabetic individuals have compromised biomechanical properties and have impaired healing capabilities [22], [23], developing a preclinical load-bearing tendon injury model was necessary to study the mechanism of impaired healing. To that end, a tendon injury and repair procedure was developed in the high fat-fed C57BL/6 mouse, a classic mouse model of obesity and type 2 diabetes [24]. A needle puncture injury was evaluated and perfected to avoid the need for surgical repair of the injury and immobility of the tendon during the healing process, both common requirements in other published injury models (Figure 1A–C) [12], [25], [26]. To establish an injury that reduced tendon biomechanical properties by approximately 40–50%, tendons from 17-week-old mice were punctured *in situ* with needles of various diameters and immediately harvested. Biomechanical testing of the injured tendons showed statistically significant decreases in maximum force (Figure 1D) and work to maximum force (Figure 1E) in tendons penetrated with 23 gauge (p<0.001) and 27 gauge (p<0.01) needles when compared to the uninjured tendons. There was a significant decrease in stiffness (p<0.05) only with the 23 gauge needle (Figure 1F). Based on these findings, the 23-gauge needle was chosen for all subsequent studies.

### Biomechanical properties of the FDL tendon in obese, HF-fed and lean mice

**Figure 2:**
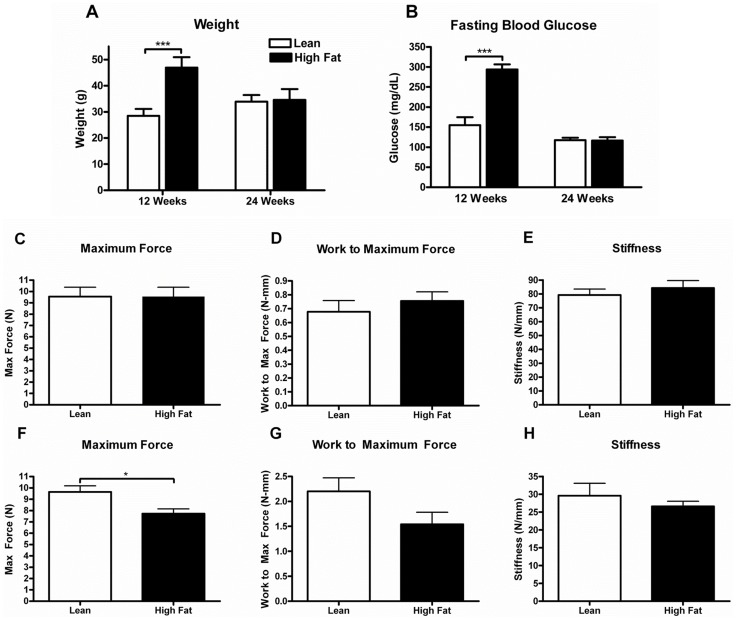
Long-term effects of high fat diet on biomechanical properties of FDL tendon in the absence of injury. Five-week-old C57BL/6 male mice were placed on HF (60% kcal from fat) or lean control diets (10% kcal from fat) for 12 weeks. After 12 weeks on diet, half of the HF-fed mice were switched to the lean diet and continued on this diet for an additional 12 weeks along with comparable numbers of mice continuously fed the lean diet. Body weight (A) and fasting glucose levels (B) were determined in all groups at the 12 and 24 week time points. Maximum force (C,F), work to maximum force (D,G) and stiffness (E,H) were measured in FDL tendons harvested at 12 weeks (C,D,E) and 24 weeks (F,G,H) from each of the diet groups. Results are represented as mean ± SEM (n = 7 at 12 weeks and n = 5–6 at 24 weeks). *** p<0.001 and * p<0.05.

Male C57BL/6 mice were placed on a HF (60% kcal) or low fat (10% kcal) diet for 12 weeks. The high fat diet resulted in a 65% increase in body weight over the lean controls (46.9±.7 g versus 28.5±.4 g; p<.001; Figure 2A). Fasting blood glucose levels were 90% higher in the HF diet group when compared to lean (294.0±32.8 mg/dl versus 155.0±53.2 mg/dl; p<.001; Figure 2B), confirming the hyperglycemia and metabolic dysfunction of this mouse model of type 2 diabetes. Reversal of the obesity and associated metabolic dysfunction was observed after placing HF-fed mice back onto the lean diet for a subsequent 12 weeks (Figure 2A,B).

Biomechanical analyses were performed on FDL tendons from HF-fed and lean mice in the absence of injury to determine if obesity and diabetes compromised tendon biomechanical properties. After an initial 12 weeks on diet, no significant difference in biomechanical parameters was observed between mice on the HF and lean diets (Figure 2C–E). In contrast, a significant decrease in maximum force was observed in tendons from HF-fed mice relative to the lean diet-fed mice after 12 subsequent weeks on the lean diet (Figure 2F–H).

### Tendon repair in a mouse model of type 2 diabetes

**Figure 3:**
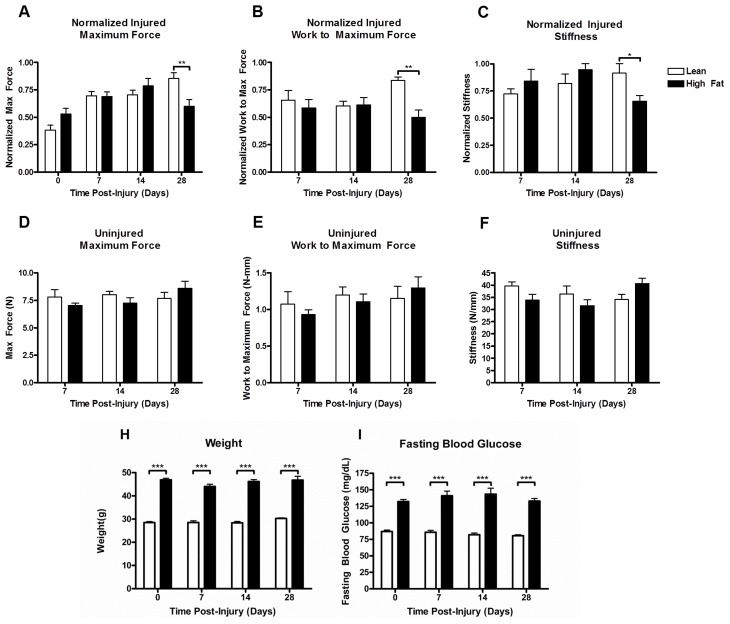
Effect of diet on biomechanical properties of the FDL tendon during injury repair. Five-week-old C57BL/6 male mice were placed on HF (60% kcal from fat) or lean control diets (10% kcal from fat) for 12 weeks. Following stab injury, mice were maintained on their respective diets until sacrifice. FDL tendons were harvested at the time points indicated and biomechanical properties determined. Maximum force (A, D), work to maximum force (B, E) and stiffness (C, F) were measured in injured (A,B,C) and contralateral control tendons (D,E,F). Values for injured tendons were normalized to the respective contralateral tendons at 7,14 and 28 days. Results are represented as mean ± SEM (n = 6–8). Body weight (H) and fasting blood glucose (I) were measured at the time of injury and again at tissue harvesting. Results are represented as mean ± SEM (n = 9–10). *** p<0.001, ** p<0.01 and * p<0.05.

After 12 weeks of HF or lean diet feeding, mice were subjected to the right FDL tendon puncture injury. Biomechanical properties of tendons were assessed over the subsequent 28 days as a function of diet. The maximum force, work to maximum force, and stiffness for each injured tendon were normalized to values determined from the respective contralateral tendon. Biomechanical properties of FDL tendons from the HF-fed mice were significantly compromised relative to values from lean-fed mice as expressed by the interaction term computed from two-way ANOVA (p<0.05). Additionally, from post-hoc analysis, statistically significant differences were observed in normalized maximum force (p<0.01; Figure 3A), normalized work to maximum force (p<0.01; Figure 3B), and normalized stiffness (p<0.05; Figure 3C) at 28 days between HF-fed and lean-fed mice. Consistent with our earlier data, sham operated tendons showed no significant differences in biomechanical parameters between diet groups.

Weight and fasting glucose levels were monitored in the injured mice over the course of the 28 day repair period. No changes in either weight or glucose levels were detected in mice on the HF or lean diets.

### Histologic analysis of tendon repair following puncture injury

**Figure 4:**
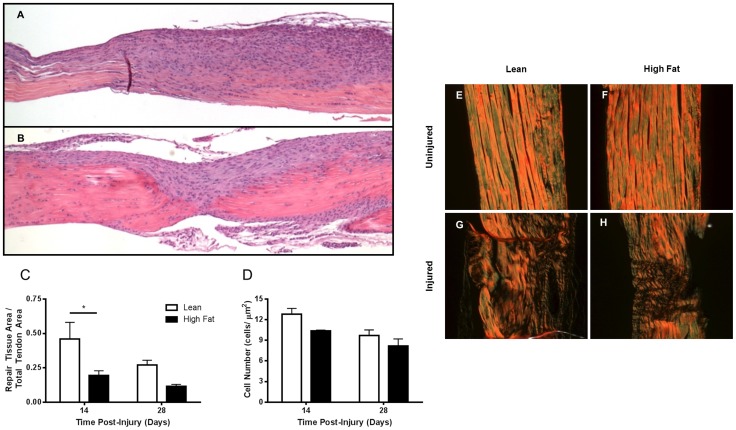
Effect of high fat diet on tissue repair, cellular recruitment and collagen organization/fiber alignment during the FDL tendon repair process. Mice were placed on HF or lean control diets for 12 weeks. Following stab injury, mice were maintained on their respective diets until sacrifice. FDL tendons were harvested at 7 day intervals for 28 days and processed for histologic analysis. Longitudinal sections of injured FDL tendons at day 14 from mice on lean (A) or HF (B) diets were stained with hematoxylin-eosin (5× magnification). Area of repair tissue (increased cellularity) was quantitated as a fraction of total tendon area (C). The number of cells per unit area of repair tissue was determined (D). Longitudinal sections of uninjured (E,F) and injured FDL (G–H) tendons at day 28 were stained with picrosirius red and visualized under polarized light (20× magnification). Brighter color intensities indicate organized and aligned collagen fibers while zones of disorganized or absent collagen appear dark. Results are represented as mean ± SEM (n = 2–4). * p<0.05.

The results of biomechanical testing of the FDL tendon following puncture injury argue for a detrimental effect of HF-feeding and the associated obesity and type 2 diabetes on this process. To assess the repair process at the tissue and cellular level, a histologic examination of the tendons at 14 and 28 days post-injury was performed. The most apparent difference in the two dietary treatments was the consistently smaller cellular and fibrous repair tissue at the injury site at both time points in the high fat diet-fed mice (Figure 4A–D). The repair tissue area fraction was significantly reduced in the HF-fed compared to LF-fed mice at day 14, and tended to continue to be reduced at day 28 as well (Figure 4C). Cellularity in the repair tissue was also lower in the HF-fed mice at the 14 and 28-day time points, albeit these differences were not statistically significant (Figure 4D). Visualizing picrosirius red-stained sections under polarized light revealed the degree of collagen fiber alignment in the zone of repair and in the surrounding uninjured tendon (Figure 4E–H). The degree of collagen remodeling and fiber alignment at day 28 in the injured area of the tendon was less in the HF mice relative to lean controls (Figure 4G, H). This is consistent with compromised biomechanical properties at that time point and is associated with decreased repair tissue size and cellularity.

### Insulin receptor expression in cells at the site of tendon injury repair

It has been reported that tendon injury repair is defective in obese, type 2 diabetic patients [27]. Data presented here confirm these observations in a classic mouse model of obesity and type 2 diabetes. It is not known, however, whether insulin resistance of obesity and diabetes is directly affecting the cells involved in the tendon healing process or affects healing indirectly through the associated hyperglycemia and other metabolic dysfunction that are characteristic of this condition. To begin to address this question, the expression of insulin receptors on the cells in the healing tendon was assessed.

**Figure 5:**
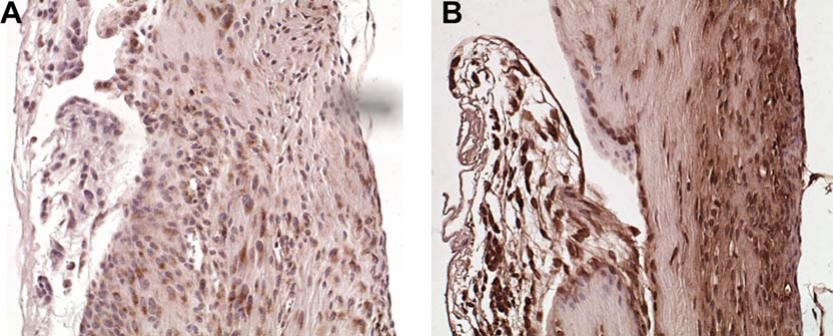
Insulin receptors are expressed by cells involved in the FDL tendon repair process. Longitudinal sections of FDL tendons from lean (A) and HF (B) diet-fed mice at day 28 post-injury were stained for insulin receptors (20× magnification). Strong positive staining (brown) was present in the majority of cells in the repair zone regardless of dietary treatment.

Immunohistochemical staining with an anti-insulin receptor antibody demonstrated that cells at the tendon repair site expressed abundant insulin receptors in both HF-fed and lean mice (Figure 5A,B). This positivity was observed at all-time points during the repair process and in both the injured and uninjured tendons (data not shown). These findings suggest that insulin may potentially have direct effects on the tendon healing process as well as on normal tendon metabolic homeostasis.

## Discussion

Diabetic patients are at increased risk for tendinopathies [28]. The prevalence of these conditions in both type 1 and type 2 diabetes increases with duration of the disease and may be linked to hyperglycemia [28]. Several clinical case reports describe thickened, fibrotic tendons and decreased range of motion in diabetic patients [22], [23], [29]. Importantly, these patients are also at increased risk for tendon rupture and have less satisfactory outcomes following surgical repair [28], [30]. In the current study, biomechanical testing in injured FDL tendons from a HF diet-induced obese, type 2 diabetic mouse model demonstrates the detrimental effect of this metabolic state on tendon repair. Biomechanical properties of repaired tendons from these obese, diabetic mice are inferior to those of lean control mice. The difference in biomechanical properties of the two populations, however, was only statistically significant at day 28 post injury. This suggests that the deficiencies in the tendon repair process in HF diet-induced obese, diabetic mice were only manifest in the later stages of repair, perhaps during the remodeling phase. The assessment of collagen organization and alignment using picrosirius red staining with visualization under plain polarized light supports this contention that remodeling is delayed or impaired in this model of HF diet-induced obesity and type 2 diabetes.

As stated above, tendons from diabetic patients tend to be abnormal in parameters of strength and functionality. Results of current investigations were somewhat surprising in that biomechanical properties of the FDL tendon in the absence of injury were not compromised in mice after 12 weeks on a high fat diet. However, these parameters were subsequently decreased when HF-fed mice were placed on lean chow diets for an additional 12 weeks and compared to mice maintained for similar periods of time exclusively on a lean chow diet. These results may be consistent with clinical observations indicating that risk for tendinopathies in diabetic patients is associated with duration of disease [31], [32]. While switching from HF to lean chow diets reverses obesity and diabetes over the course of 12 weeks, some pathologic changes that are initiated during the HF-feeding stage are expected to be retained if not increased, though at progressively decreasing intensity over the subsequent 12 week period as metabolic dysfunction corrects. These cumulative changes to the tendon may ultimately reach levels that compromise biomechanical properties to a degree that can be detected by our experimental methods.

While cellular abnormalities in tendon repair have not previously been characterized in models of type 2 diabetes, Chbinou et. al. [33] have described decreased inflammatory cellular infiltrates and decreased cellular proliferation in response to tendon injury in a streptozotocin-induced type 1 diabetic rat model. The results in the current report also document decreased cellular recruitment to or proliferation at the tendon injury site resulting in smaller scar tissue area. Appreciating the many important differences in type 1 and type 2 diabetes, the common characteristics of hyperglycemia and decreased cellular insulin signaling are potential mediators of the cellular abnormalities that are observed in both. The similarities in abnormal tendon repair between models of type 1 diabetes and our HF diet-induced type 2 diabetes also support the conclusion that it is the metabolic dysfunction of diabetes not the high fat diet alone that is responsible for defects in tendon repair. Additional investigations, however, will be required to address the relative contributions of HF diet alone versus the interrelated metabolic dysfunction of obesity and type 2 diabetes on tendon repair.

The natural aging process is well known to negatively affect collagen-containing tissues such as tendon, in part through the accumulation of advanced glycation end-products (AGE) which crosslink collagen molecules [34]. The hyperglycemia of diabetes is associated with accelerated glycation of proteins and an increased accumulation of AGE crosslinking in long-lived proteins such as collagen [35], [36]. In regards to tendon integrity, crosslinking from glycation induces an expansion of the molecular packing and also covalently crosslinks collagen to soluble plasma proteins [37]. The pathological crosslink formation induced by AGE leads to increased stiffness of the protein matrix, hence impeding function as well as increasing resistance to removal by proteolytic means, which in turn affects the process of tissue remodeling [38]. Thus, it is logical to propose that diabetics with hyperglycemia and accelerated changes in collagen structure contribute to compromised tendon integrity and an impaired tendon healing process.

While hyperglycemia has received most attention when mechanisms mediating tendinopathies in diabetes are discussed, our observation that insulin receptors are expressed in most cells at the site of tendon repair argues for a potentially direct effect of insulin on tendon repair that may be disrupted by insulin deficiency (type 1 diabetes) or insulin resistance (type 2 diabetes). Future work must identify the cells in the tendon repair process whose numbers and/or functions are compromised in diabetes. Determining the mechanism of the diabetes-dependent dysfunction will also be critical in addressing potential therapeutic interventions to promote tendon healing.

## References

[1] Zimmet P, Alberti KG, Shaw J (2001) Global and societal implications of the diabetes epidemic. Nature 414: 782–787. 11742409 10.1038/414782a

[2] Taylor R (2012) Insulin resistance and type 2 diabetes. Diabetes 61: 778–779. 22442298 10.2337/db12-0073 PMC3314346

[3] (2011) Centers for Disease Control and Prevention. National Diabetes Fact Sheet, 2011.

[4] Bloomgarden ZT (2000) American Diabetes Association Annual Meeting, 1999: diabetes and obesity. Diabetes Care 23: 118–124. 10857981 10.2337/diacare.23.1.118

[5] American Diabetes A (2013) Economic costs of diabetes in the U.S. in 2012. Diabetes Care 36: 1033–1046. 23468086 10.2337/dc12-2625 PMC3609540

[6] Boyle JP, Thompson TJ, Gregg EW, Barker LE, Williamson DF (2010) Projection of the year 2050 burden of diabetes in the US adult population: dynamic modeling of incidence, mortality, and prediabetes prevalence. Popul Health Metr 8: 29. 20969750 10.1186/1478-7954-8-29 PMC2984379

[7] Huang ES, Basu A, O'Grady M, Capretta JC (2009) Projecting the future diabetes population size and related costs for the U.S. Diabetes Care 32: 2225–2229. 19940225 10.2337/dc09-0459 PMC2782981

[8] Voleti PB, Buckley MR, Soslowsky LJ (2012) Tendon healing: repair and regeneration. Annu Rev Biomed Eng 14: 47–71. 22809137 10.1146/annurev-bioeng-071811-150122

[9] James R, Kesturu G, Balian G, Chhabra AB (2008) Tendon: biology, biomechanics, repair, growth factors, and evolving treatment options. J Hand Surg Am 33: 102–112. 18261674 10.1016/j.jhsa.2007.09.007

[10] Wojciak B, Crossan JF (1993) The accumulation of inflammatory cells in synovial sheath and epitenon during adhesion formation in healing rat flexor tendons. Clin Exp Immunol 93: 108–114. 8324895 10.1111/j.1365-2249.1993.tb06505.x PMC1554749

[11] Sharma P, Maffulli N (2005) Tendon injury and tendinopathy: healing and repair. J Bone Joint Surg Am 87: 187–202. 15634833 10.2106/JBJS.D.01850

[12] Palmes D, Spiegel HU, Schneider TO, Langer M, Stratmann U, et al (2002) Achilles tendon healing: long-term biomechanical effects of postoperative mobilization and immobilization in a new mouse model. J Orthop Res 20: 939–946. 12382957 10.1016/S0736-0266(02)00032-3

[13] Branford OA, Mudera V, Brown RA, McGrouther DA, Grobbelaar AO (2008) A novel biomimetic material for engineering postsurgical adhesion using the injured digital flexor tendon-synovial complex as an in vivo model. Plast Reconstr Surg 121: 781–793. 18317128 10.1097/01.prs.0000299373.25294.65

[14] Garner WL, McDonald JA, Koo M, Kuhn C 3rd, Weeks PM (1989) Identification of the collagen-producing cells in healing flexor tendons. Plast Reconstr Surg 83: 875–879. 2652163 10.1097/00006534-198905000-00018

[15] Chuen FS, Chuk CY, Ping WY, Nar WW, Kim HL, et al (2004) Immunohistochemical characterization of cells in adult human patellar tendons. J Histochem Cytochem 52: 1151–1157. 15314082 10.1369/jhc.3A6232.2004

[16] Loiselle AE, Bragdon GA, Jacobson JA, Hasslund S, Cortes ZE, et al (2009) Remodeling of murine intrasynovial tendon adhesions following injury: MMP and neotendon gene expression. J Orthop Res 27: 833–840. 19051246 10.1002/jor.20769 PMC4472455

[17] Bedi A, Fox AJ, Harris PE, Deng XH, Ying L, et al (2010) Diabetes mellitus impairs tendon-bone healing after rotator cuff repair. J Shoulder Elbow Surg 19: 978–988. 20303293 10.1016/j.jse.2009.11.045 PMC5257255

[18] Ramchurn N, Mashamba C, Leitch E, Arutchelvam V, Narayanan K, et al (2009) Upper limb musculoskeletal abnormalities and poor metabolic control in diabetes. Eur J Intern Med 20: 718–721. 19818294 10.1016/j.ejim.2009.08.001

[19] Gaida JE, Cook JL, Bass SL (2008) Adiposity and tendinopathy. Disabil Rehabil 30: 1555–1562. 18608380 10.1080/09638280701786864

[20] Hasslund S, Jacobson JA, Dadali T, Basile P, Ulrich-Vinther M, et al (2008) Adhesions in a murine flexor tendon graft model: autograft versus allograft reconstruction. J Orthop Res 26: 824–833. 18186128 10.1002/jor.20531 PMC2709286

[21] Elfar JC, Jacobson JA, Puzas JE, Rosier RN, Zuscik MJ (2008) Erythropoietin accelerates functional recovery after peripheral nerve injury. J Bone Joint Surg Am 90: 1644–1653. 18676893 10.2106/JBJS.G.00557 PMC4470043

[22] Chen AL, Shapiro JA, Ahn AK, Zuckerman JD, Cuomo F (2003) Rotator cuff repair in patients with type I diabetes mellitus. J Shoulder Elbow Surg 12: 416–421. 14564259 10.1016/s1058-2746(03)00172-1

[23] Akturk M, Ozdemir A, Maral I, Yetkin I, Arslan M (2007) Evaluation of Achilles tendon thickening in type 2 diabetes mellitus. Exp Clin Endocrinol Diabetes 115: 92–96. 17318767 10.1055/s-2007-955097

[24] Surwit RS, Kuhn CM, Cochrane C, McCubbin JA, Feinglos MN (1988) Diet-induced type II diabetes in C57BL/6J mice. Diabetes 37: 1163–1167. 3044882 10.2337/diab.37.9.1163

[25] Loiselle AE, Bragdon GA, Jacobson JA, Hasslund S, Cortes ZE, et al (2009) Remodeling of murine intrasynovial tendon adhesions following injury: MMP and neotendon gene expression. Journal of Orthopaedic Research 27: 833–840. 19051246 10.1002/jor.20769 PMC4472455

[26] Gelberman RH, Botte MJ, Spiegelman JJ, Akeson WH (1986) The excursion and deformation of repaired flexor tendons treated with protected early motion. J Hand Surg Am 11: 106–110. 3944424 10.1016/s0363-5023(86)80115-0

[27] Clement ND, Hallett A, MacDonald D, Howie C, McBirnie J (2010) Does diabetes affect outcome after arthroscopic repair of the rotator cuff? J Bone Joint Surg Br 92: 1112–1117. 20675756 10.1302/0301-620X.92B8.23571

[28] Abate M, Schiavone C, Salini V, Andia I (2013) Occurrence of tendon pathologies in metabolic disorders. Rheumatology (Oxford) 52: 599–608. 23315787 10.1093/rheumatology/kes395

[29] Ramirez LC, Raskin P (1998) Diabetic foot tendinopathy: abnormalities in the flexor plantar tendons in patients with diabetes mellitus. J Diabetes Complications 12: 337–339. 9877468 10.1016/s1056-8727(98)00024-5

[30] DiDomenico LA, Williams K, Petrolla AF (2008) Spontaneous rupture of the anterior tibial tendon in a diabetic patient: results of operative treatment. J Foot Ankle Surg 47: 463–467. 18725129 10.1053/j.jfas.2008.05.007

[31] Rosenbloom AL, Silverstein JH (1996) Connective tissue and joint disease in diabetes mellitus. Endocrinol Metab Clin North Am 25: 473–483. 8799711 10.1016/s0889-8529(05)70335-2

[32] Aydeniz A, Gursoy S, Guney E (2008) Which musculoskeletal complications are most frequently seen in type 2 diabetes mellitus? J Int Med Res 36: 505–511. 18534132 10.1177/147323000803600315

[33] Chbinou N, Frenette J (2004) Insulin-dependent diabetes impairs the inflammatory response and delays angiogenesis following Achilles tendon injury. Am J Physiol Regul Integr Comp Physiol 286: R952–957. 14715491 10.1152/ajpregu.00536.2003

[34] Vlassara H, Bucala R, Striker L (1994) Pathogenic effects of advanced glycosylation: biochemical, biologic, and clinical implications for diabetes and aging. Lab Invest 70: 138–151. 8139257

[35] Singh R, Barden A, Mori T, Beilin L (2001) Advanced glycation end-products: a review. Diabetologia 44: 129–146. 11270668 10.1007/s001250051591

[36] Brownlee M (2001) Biochemistry and molecular cell biology of diabetic complications. Nature 414: 813–820. 11742414 10.1038/414813a

[37] Tanaka S, Avigad G, Brodsky B, Eikenberry EF (1988) Glycation induces expansion of the molecular packing of collagen. J Mol Biol 203: 495–505. 3143838 10.1016/0022-2836(88)90015-0

[38] Reddy GK (2004) Cross-linking in collagen by nonenzymatic glycation increases the matrix stiffness in rabbit achilles tendon. Exp Diabesity Res 5: 143–153. 15203885 10.1080/15438600490277860 PMC2496877

